# Activation of ERα Signaling Differentially Modulates IFN-γ Induced HLA-Class II Expression in Breast Cancer Cells

**DOI:** 10.1371/journal.pone.0087377

**Published:** 2014-01-27

**Authors:** Ahmed A. Mostafa, Dianne Codner, Kensuke Hirasawa, Yumiko Komatsu, Matthew N. Young, Viktor Steimle, Sheila Drover

**Affiliations:** 1 Division of BioMedical Sciences, Immunology and Infectious Diseases Research Group, Faculty of Medicine, Memorial University of Newfoundland, St. John's, Newfoundland and Labrador, Canada; 2 Département de Biologie, Université de Sherbrooke, Sherbrooke, Québec, Canada; Oklahoma Medical Research Foundation, United States of America

## Abstract

The coordinate regulation of HLA class II (HLA-II) is controlled by the class II transactivator, CIITA, and is crucial for the development of anti-tumor immunity. HLA-II in breast carcinoma is associated with increased IFN-γ levels, reduced expression of the estrogen receptor (ER) and reduced age at diagnosis. Here, we tested the hypothesis that estradiol (E_2_) and ERα signaling contribute to the regulation of IFN-γ inducible HLA-II in breast cancer cells. Using a panel of established ER^−^ and ER^+^ breast cancer cell lines, we showed that E_2_ attenuated HLA-DR in two ER^+^ lines (MCF-7 and BT-474), but not in T47D, while it augmented expression in ER^−^ lines, SK-BR-3 and MDA-MB-231. To further study the mechanism(s), we used paired transfectants: ERα^+^ MC2 (MDA-MB-231 c10A transfected with the wild type ERα gene) and ERα^−^ VC5 (MDA-MB-231 c10A transfected with the empty vector), treated or not with E_2_ and IFN-γ. HLA-II and CIITA were severely reduced in MC2 compared to VC5 and were further exacerbated by E_2_ treatment. Reduced expression occurred at the level of the IFN-γ inducible CIITA promoter IV. The anti-estrogen ICI 182,780 and gene silencing with *ESR1* siRNA reversed the E_2_ inhibitory effects, signifying an antagonistic role for activated ERα on CIITA pIV activity. Moreover, STAT1 signaling, necessary for CIITA pIV activation, and selected STAT1 regulated genes were variably downregulated by E_2_ in transfected and endogenous ERα positive breast cancer cells, whereas STAT1 signaling was noticeably augmented in ERα^−^ breast cancer cells. Collectively, these results imply immune escape mechanisms in ERα^+^ breast cancer may be facilitated through an ERα suppressive mechanism on IFN-γ signaling.

## Introduction

Antigen presentation by major histocompatibility complex (MHC) class II molecules (MHC-II), known as HLA-II (HLA-DR, -DP, -DQ) in humans and co-chaperones HLA-DM and the invariant chain (Ii) are important for the development of adaptive immune responses including anti-tumor immunity [1]–[4]. Typically, HLA-II expression is limited to professional antigen presenting cells (pAPC), but is induced by IFN-γ on most cell types including those derived from cancer [5], [6]. HLA-DR positive tumor cells have been described in several malignancies, such as melanoma [7], colon [8], [9] and breast [10]–[12], but the underlying mechanisms are likely diverse. The number of HLA-II positive tumor cells in breast cancer is directly associated with tumor infiltrating immune cells and levels of IFN-γ [12]–[14], but other cytokines, hormones, growth factors and oncogenes are also implicated in regulating HLA-II expression [15]–[20].

HLA-II expression is controlled at the transcription level by a highly conserved regulatory module, located in the promoter of genes encoding the α- and β-chains of all HLA-II molecules and in the gene encoding the Ii co-chaperone [21]–[26]. This regulatory module forms a platform for the class II transactivator (CIITA), a non-DNA binding protein, which acts as a transcriptional integrator by connecting transcription factors, bound to the MHC-II promoter with components of the general transcriptional machinery [23], [27]–[30]. The central role of CIITA is evident from lack of constitutive or IFN-γ inducible HLA-II in bare lymphocyte syndrome [31], [32].

CIITA expression is controlled by three distinct promoters: promoter I (pI) for constitutive expression in dendritic cells; promoter III (pIII), for constitutive expression in B cells; promoter IV (pIV) for IFN-γ inducible expression [21], [26], [33]. This promoter system is crucial for controlling CIITA messenger RNA (mRNA) and protein levels, and they, in turn, regulate HLA-II expression. The molecular regulation of CIITA pIV is intricately linked to the classical IFN-γ signaling pathway. IFN-γ, binds to IFN-γ receptors (IFNGR) on the cell surface, resulting in autophosphorylation of Janus kinase 2 (JAK2) and JAK1, followed by phosphorylation, dimerization and nuclear translocation of signal transducer and activator of transcription 1 (STAT1) [34], [35]. Phosphorylated STAT1 (pSTAT1) binds to IFN-activated sites (GAS) in the promoter of target genes including the IFN-regulatory factor 1 (IRF1), thus stimulating its expression. IRF1 binds cooperatively with IRF2 to its associated IRF element (IRF-E) in CIITA pIV, and concomitant pSTAT1 binding to GAS in CIITA pIV results in transcriptional activation of CIITA [33], [36]. Moreover, signaling pathways such as mitogen activated protein kinases (MAPK) and PI3K/Akt that are frequently activated in breast cancer cells [37] modulate expression of IRF1 and STAT1 [38]–[40], further impacting the levels of IFN-γ inducible CIITA and subsequent HLA-II expression on tumor cells.

Previously, we showed that HLA-II (HLA-DR, HLA-DM and Ii) was discordantly expressed on tumor cells in human breast cancer tissues [12]. Furthermore, tumor cell expression of HLA-DR and Ii, but not HLA-DM, correlated with reduced expression of estrogen receptors (ER) and reduced age at diagnosis. Importantly, tumors with coordinate expression of HLA-DR, Ii and HLA-DM had the highest IFN-γ mRNA levels and correlated with increased patient survival [12]. Undoubtedly, the mechanisms governing tumor cell expression of HLA-II in breast carcinoma are likely multifaceted, involving IFN-γ secreted by infiltrating immune cells [12], circulating and tumor-associated estrogens [41] and activation of growth factor and hormone receptor pathways in the tumor cells [42], [43]. Estradiol and anti-estrogens, tamoxifen and fulvestrant or ICI 180,720 (ICI), were shown to modulate IFN-γ inducible MHC-II in various cell types [17], [19], [44], [45] through mechanisms not involving ligand activation of the estrogen receptor (ER) pathway.

In this study, using established human ER^−^ and ER^+^ breast cancer cell lines (BCCL) and an ERα-transfected BCCL, we investigated the specific and combined effects of estradiol (E_2_) and ERα on HLA-II regulation. We found IFN-γ inducible HLA-II expression was modulated by E_2_-ER activation at the level of the CIITA pIV. Furthermore, E_2_-treatment of ERα^+^ BCCL and ERα^−^ BCCL differentially affected various components of the IFN-γ signaling pathway that are required for transactivation of CIITA pIV.

## Results

### Estradiol differentially modulates HLA-DR expression in breast cancer cell lines

**Figure 1:**
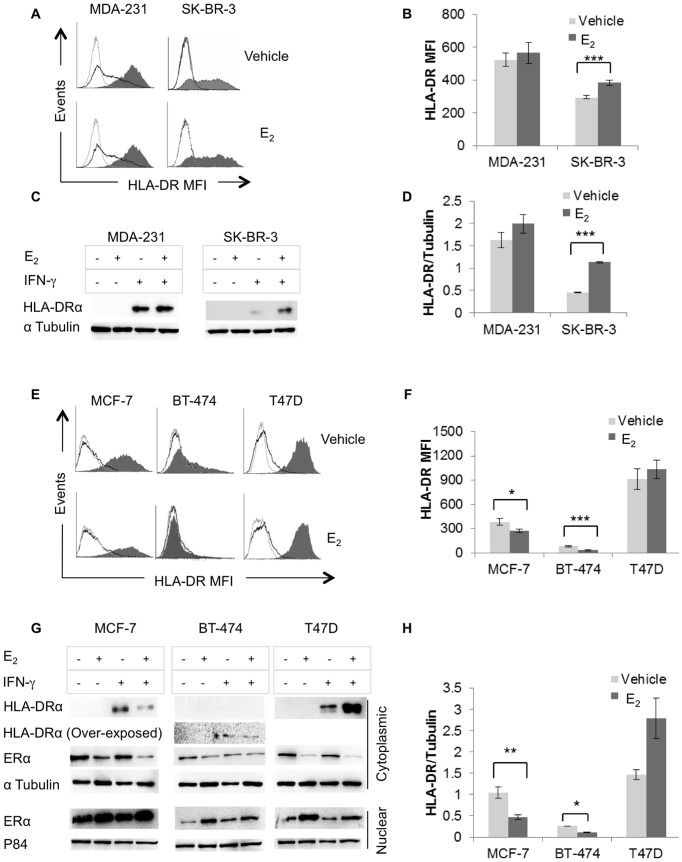
E_2_ differentially modulates inducible HLA-DR expression in ERα^+^ and ERα^−^ breast cancer cell lines. MDA-MB-231, SK-BR-3, MCF-7, BT-474, and T47D were cultured in E_2_-depleted media, treated with vehicle (ethanol) or E_2_ (10^−9^ M) and stimulated or not with IFN-γ (100 U/ml) for 96 hours. (A & E) HLA-DR cell surface expression (L243) was analyzed by flow cytometry: grey line, isotype control; black line, constitutive expression; shaded histogram, IFN-γ induced expression. (B & F) Bar graphs represent the MFI (mean florescence intensity) ± SEM for HLA-DR expression of three independent experiments. (C & G) Western blot analysis was performed on cytoplasmic and nuclear extracts for ERα expression (HC-20) and on cytoplasmic extracts for HLA-DRα (TAL 1B5). Protein loading controls included α-tubulin (B-7) and P84 (5E10) for cytoplasmic and nuclear proteins, respectively. (D & H) Bar graphs show the ratio of band intensity for HLA-DRα, normalized to the α-tubulin band intensity and represent the mean ± SEM of three independent experiments (*p<0.05, **p<0.01, ***p<0.001).

Stemming from our previous finding that HLA-II expression in breast carcinoma tissues correlates with increased IFN-γ mRNA, reduced age at diagnosis and reduced ER levels [12] we questioned whether E_2_, in the absence or presence of its cognate receptor ERα, modulates HLA-DR expression in established ER^−^ and ER^+^ BCCL, treated or not with IFN-γ for 96 hours. Analysis of ER^−^ BCCL using flow cytometry (Figure 1A & 1B) revealed low basal expression of HLA-DR in MDA-MB-231, but not in SK-BR-3 while IFN-γ induced strong expression in both cell lines. E_2_-treatment augmented IFN-γ inducible HLA-DR, although this was significant for only SK-BR-3 (Figure 1B). These results, confirmed by Western blot analysis of cell lysates (Figure 1C & 1D), suggest E_2_ may modulate HLA-DR expression in ER^−^ breast cancer through an ERα independent mechanism [46].

Since the least HLA-DR in human breast carcinoma tissues occurred in ER^+^ tumors [12] we hypothesized that E_2_-activation of the ERα pathway inhibits HLA-DR expression. Analysis of ER^+^ BCCL, treated as described above, revealed a variable pattern of IFN-γ inducible HLA-DR expression with amounts that were barely detectable, moderate and abundant in BT-474, MCF-7, and T47D, respectively (Figure 1E & 1F). Constitutive HLA-DR was detected at the cell surface in only T47D (Figure 1E). Furthermore, E_2_ treatment significantly reduced HLA-DR in MCF-7 and BT-474, but not in T47D (Figure 1E). Similar results were obtained from Western blot analysis of cell lysates (Figure 1G & 1H). Notably, ERα levels were not altered by IFN-γ but E_2_ treatment increased the amount in the nucleus, indicating ligand activation of the ERα pathway (Figure. 1G). Taken together these data suggest that E_2_-inhibition of HLA-II expression in ERα^+^ BCCL is mediated through activation of ligand-dependent ERα pathway.

### Transfection of *ESR1* in an ER^-^ cell line diminishes IFN-γ inducible HLA-II proteins

**Figure 2:**
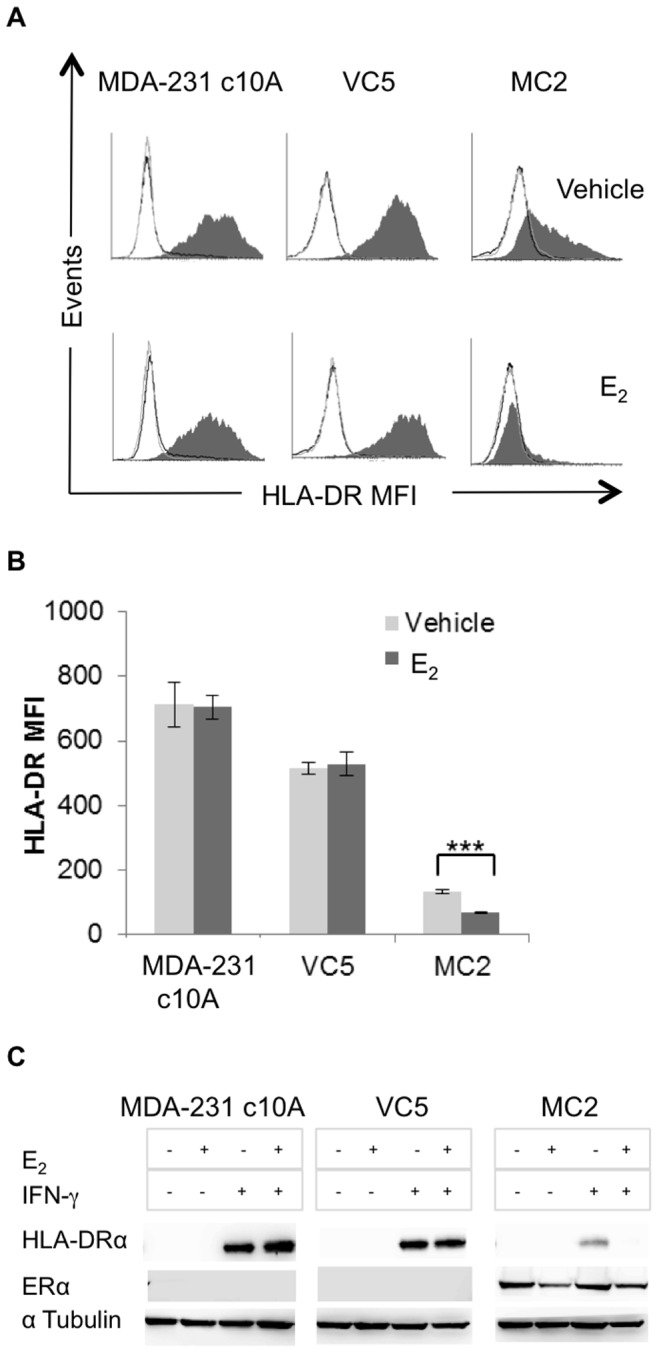
IFN-γ inducible HLA-DR is down regulated in the ERα^+^ transfected breast cancer cell line, MC2. MDA-MB-231 clone 10A (MDA-231 c10A), VC5 (MDA-231 c10A, transfected with the empty plasmid vector) and MC2 (MDA-231 c10A, transfected with wild type *ESR1*) were cultured in E_2_-depleted medium and stimulated or not with IFN-γ (100 U/ml) for 96 hours. (A) HLA-DR cell surface expression (L243) was analyzed by flow cytometry: grey line, isotype control; black line, constitutive expression; shaded histogram, IFN-γ induced expression. (B) Bar graphs represent the MFI ± SEM for HLA-DR expression of three independent experiments (***p<0.001). (C) Western blot analysis was performed on whole cell lysates for HLA-DRα (TAL 1B5) and ERα (HC-20).

To further explore the role of ERα on IFN-γ inducible HLA-DR, we used two stably transfected cell lines, derived from MDA-MB-231 clone 10A [47], [48]: MC2 expresses wild type ERα and VC5 expresses the empty vector. Since MDA-MB-231 clone 10A was selected for negative expression of ERα and ERβ [47], the transfected pair is a suitable model to assess ERα mediated effects on HLA-II without interference from other ERs including GPR30, reported to be deficient in MDA-MB-231 [48], [49]. The cells, treated and analyzed for HLA-DR expression as described above, revealed significantly reduced cell surface HLA-DR in MC2, as compared to VC5 and MDA-MB-231 clone 10A (Figure 2A & 2B). Moreover, E_2_-treatment greatly diminished HLA-DR in MC2 but not in VC5 and MDA-MB-231 clone 10A. These results were confirmed by Western blot analysis of cell extracts (Figure 2C). Again, HLA-DR protein in the ERα^+^ MC2 was severely reduced and exacerbated by E_2_, whereas MDA-MB-231 clone10A and VC5 expressed abundant HLA-DR in the presence and absence of E_2_. As the only known difference between MC2 and VC5 is the expression of ERα, these results further implicate ERα in negatively regulating HLA-DR expression.

**Figure 3:**
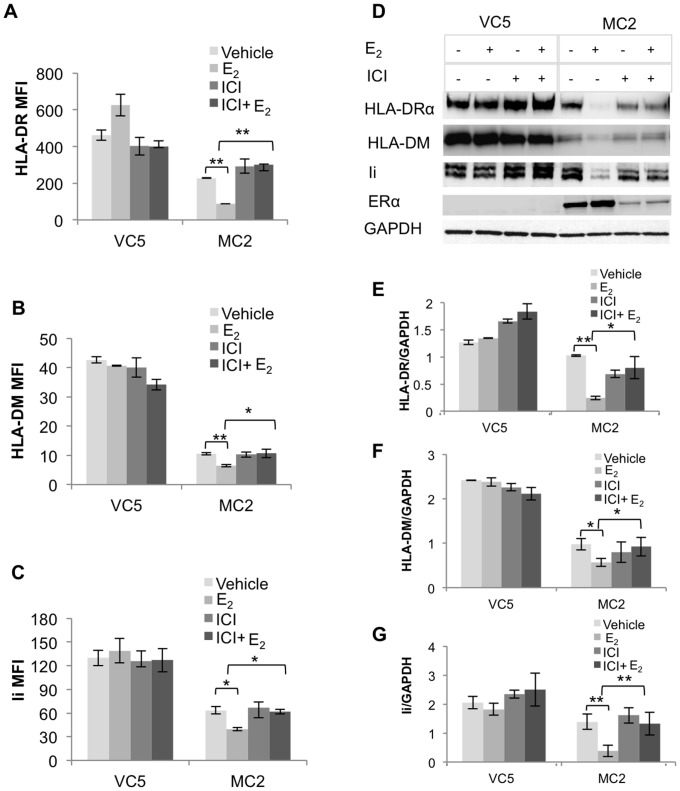
Coordinate downregulation of IFN-γ inducible HLA-II expression by E_2_ is reversed by ICI-mediated degradation of ERα in MC2 cells. VC5 and MC2 cells were cultured in E_2_-depleted media, treated with vehicle (ethanol), E_2_ (10^−9^ M) or/and ICI (10^−6^ M) followed by stimulation with IFN-γ (100 U/ml) for 96 hours. HLA-II expression was analyzed by surface flow cytometry using (A) anti-DR, (L243), and intracellular flow cytometry using (B) anti-DM (Map.DM1) and (C) anti-Ii (LN2). Bar graphs represent the MFI ± SEM of three independent experiments. (*p<0.05, **p<0.01). (D) Western blot analysis was performed on whole cell extracts using for HLA-DRα (TAL 1B5), HLA-DM (TAL18.1) and Ii (LN2); GAPDH (Ab8245) is the protein loading control. Bar graphs show the ratio of band intensities, normalized to GAPDH band intensities and represent the mean ± SEM ratio of three independent experiments: (E) HLA-DRα/GAPDH (F) HLA-DM/GAPDH, and (G) Ii/GAPDH (* p<0.05, ** p<0.01).

Although HLA-II genes are coordinately regulated [25], we found most breast cancer lesions with HLA-DR^+^ tumor cells do not have detectable HLA-DM expression [12]. We reasoned that if ERα and its activation by E_2_ coordinately down regulates HLA-II, then blocking ER signaling with ICI, a selective anti-estrogen that degrades ER, should reverse the inhibition. To test this hypothesis, MC2 and VC5 were pretreated with 10^−6^ M ICI in the presence or absence of 10^−9^ M E_2_. Following stimulation with IFN-γ for 96 hours, HLA-DR, -DM and Ii were analyzed by flow cytometry and Western blot. HLA-DR, -DM and Ii expression levels were significantly reduced in MC2 compared to VC5 (Figure 3A–3C), while E_2_-treatment further diminished HLA-II expression in MC2, but not in VC5. Although ICI-treatment, alone or with E_2_, did not restore HLA-II in MC2 to VC5 levels, it clearly reversed the E_2_-inhibitory effect on HLA-II expression. Western blot analysis (Figure 3D–3G) and immunocytochemistry (data not shown) confirmed the reduced expression of HLA-DR, -DM and Ii in MC2 and the involvement of ERα signaling in the inhibitory effect of E_2_ on HLA-II expression.

### Activation of the ERα signaling pathway impedes CIITA expression

**Figure 4:**
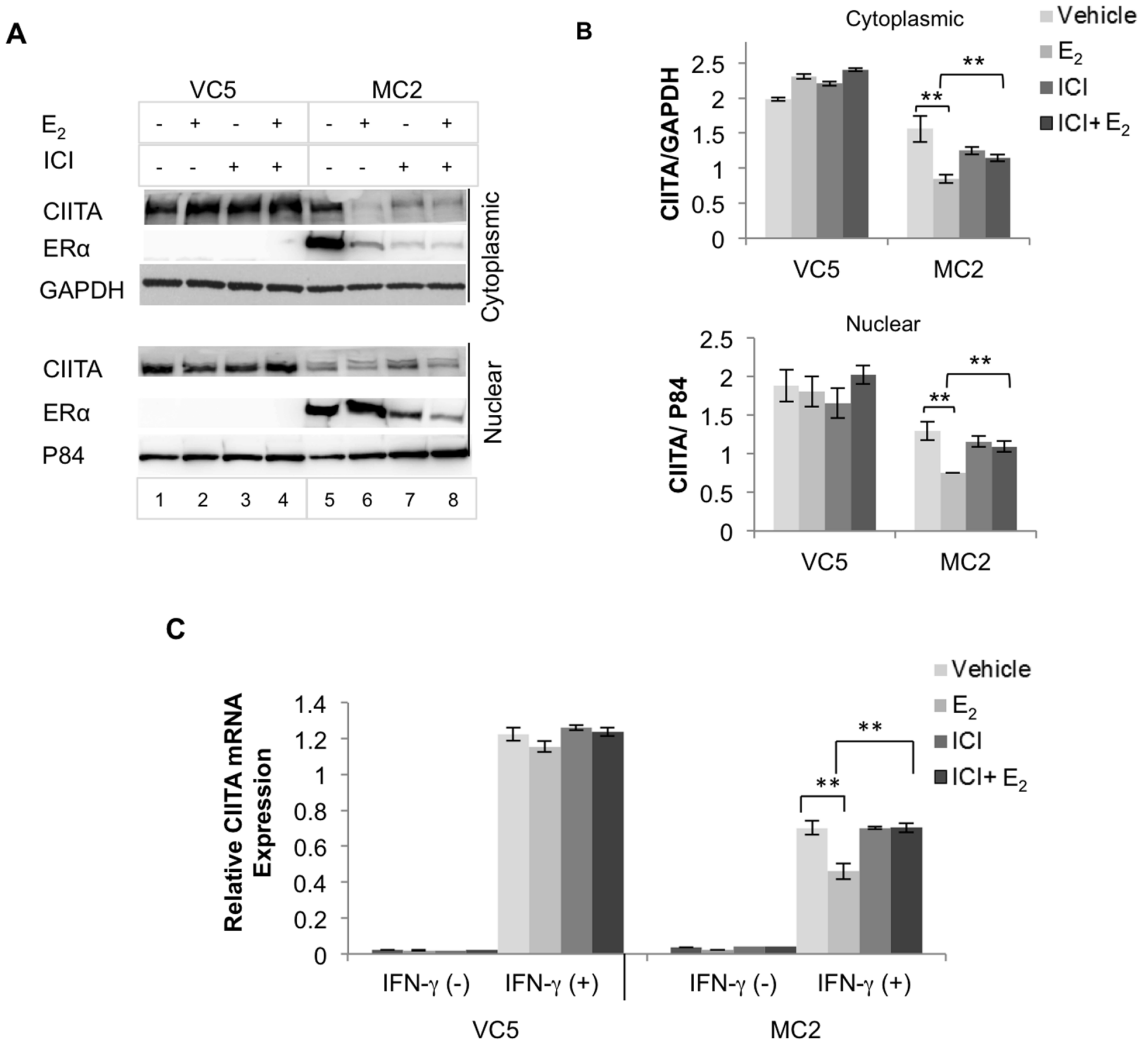
E_2_-ERα signaling down regulates CIITA protein and mRNA expression in ER^+^ BCCL. VC5 and MC2 cells were cultured in E_2_-depleted media, treated with vehicle (ethanol), E_2_ (10^−9^ M) or/and ICI (10^−6^ M) and stimulated or not with IFN-γ (100 U/ml) for 24 and 4 hours, for CIITA protein and mRNA expression, respectively. (A) Western blot analysis was performed on cytoplasmic and nuclear extracts for CIITA (antiserum #21) and ERα (HC-20). (B) Cytoplasmic CIITA and nuclear CIITA were normalized to GAPDH and P84 respectively; bar graphs represent the mean ± SEM ratio of three independent experiments (**p<0.01). (C) CIITA mRNA was relatively quantified by real time PCR using Taqman gene expression assay. GAPDH was used as an endogenous control and the data were expressed relative to a control B cell line (RAJI). Bar graphs represent the mean ± SEM of three replicate assays (**p<0.01).

Since HLA-II expression is coordinately regulated by CIITA, we predicted that ERα interfered with CIITA expression in ERα-expressing MC2. MC2 and VC5 were pretreated with E_2_ and/or ICI, as described above, followed by addition of IFN-γ for 24 hours. Western blot analysis of nuclear and cytoplasmic extracts showed inducible CIITA expression in MC2 was about 70% of VC5 levels (Figure 4A & 4B). E_2_-treatment further reduced CIITA in MC2 while increasing the amount of nuclear ERα; in contrast, ICI reversed the inhibitory effect of E_2_ on CIITA expression, coincident with ICI-mediated reduced ER levels (Fig 4A Lanes 7 and 8). These results indicated that E_2_ inhibits HLA-II expression by downregulating CIITA expression.

To further determine the inhibitory effect of E_2_ on CTIIA gene expression, VC5 and MC2 cells were pretreated with E_2_ and/or ICI for 1 hour and then stimulated with and without IFN-γ for 4 hours, an optimal time for CIITA mRNA expression [50]. CIITA transcription was induced in both VC5 and MC2, but the induction of CIITA mRNA in MC2 was about half in VC5 (Figure 4C). E_2_ further decreased CIITA mRNA in MC2, while ICI reversed the E_2_-mediated effect on CIITA.

**Figure 5:**
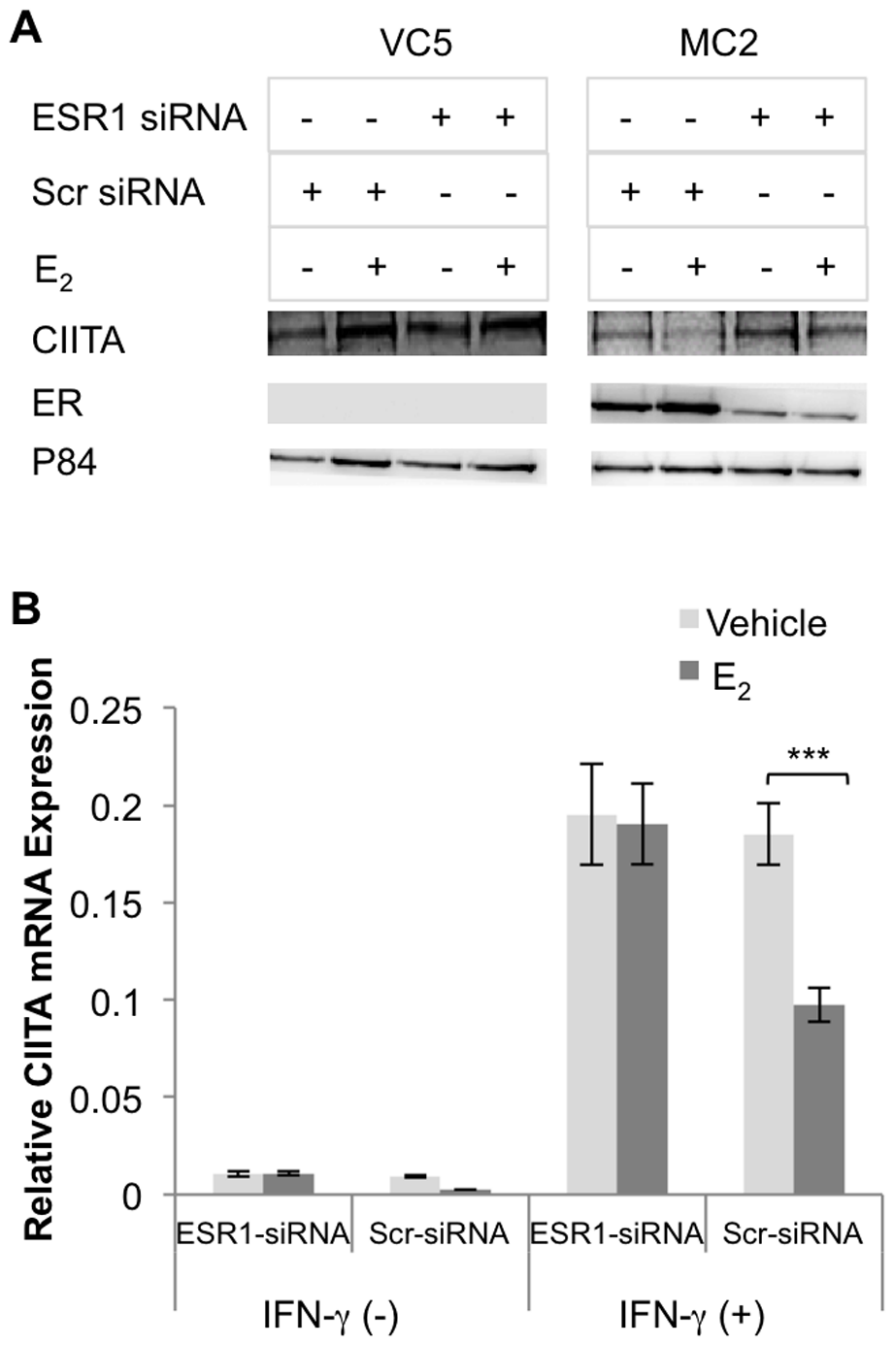
Silencing ERα with ESR1 siRNA reversed the inhibitory effect of E_2_ on CIITA expression. (A) ERα was silenced (*ESR1* siRNA) or not (scrambled siRNA) in MC2; VC5 served as an ERα negative cell control. Cells were treated with vehicle (ethanol) or E_2_ (10^−9^ M) and stimulated or not with IFN-γ (100 U/ml) for 24 hours. Nuclear lysates were prepared and probed for CIITA (anti-serum #21), ERα (HC-20), and p84. Each figure represents one of three individual experiments. (B) *ESR1* siRNA and scrambled siRNA transfected MC2 cells were treated with either vehicle (ethanol) or E_2_ (10^−9^ M) followed by stimulation with or without IFN-γ (100 U/ml) for 4 hours and CIITA mRNA was relatively quantified by real time PCR using Taqman gene expression assay. GAPDH was used as an endogenous control and the data were expressed relative to a control B cell line (RAJI). Bar graphs represent the mean ± SEM of three replicate assays (*** p<0.001).

To confirm the above results, we silenced the ERα transgene in MC2 using *ESR1* siRNA and then treated with E_2_ or vehicle control followed by IFN-γ stimulation for 24 hours. VC5, treated in the same way, was used as a control. Western blot analysis of cell lysates showed ERα was greatly reduced in MC2 transfected with ESR1 siRNA, but not with scrambled siRNA (Figure 5A). Similar to the ICI-mediated effects, ESR1 siRNA clearly reversed the E_2_-mediated inhibition observed in the scrambled siRNA transfectants. E_2_ increased CIITA in the ER^−^ VC5, whether transfected with scrambled or ESR1 siRNA. Analysis of CIITA transcripts using real time PCR on siRNA-treated cells (Figure 5B), revealed equivalent levels of CIITA transcripts in ESR1 and scrambled siRNA transfectants; again, ESR1-siRNA abolished the inhibitory effect of E_2_ on constitutive and induced CIITA transcripts. These results suggest a mechanism whereby E_2_-activated ER interferes with CIITA transcription induced by IFN-γ in breast cancer cells.

### E_2_ activated ERα inhibits CIITA promoter IV activity

**Figure 6:**
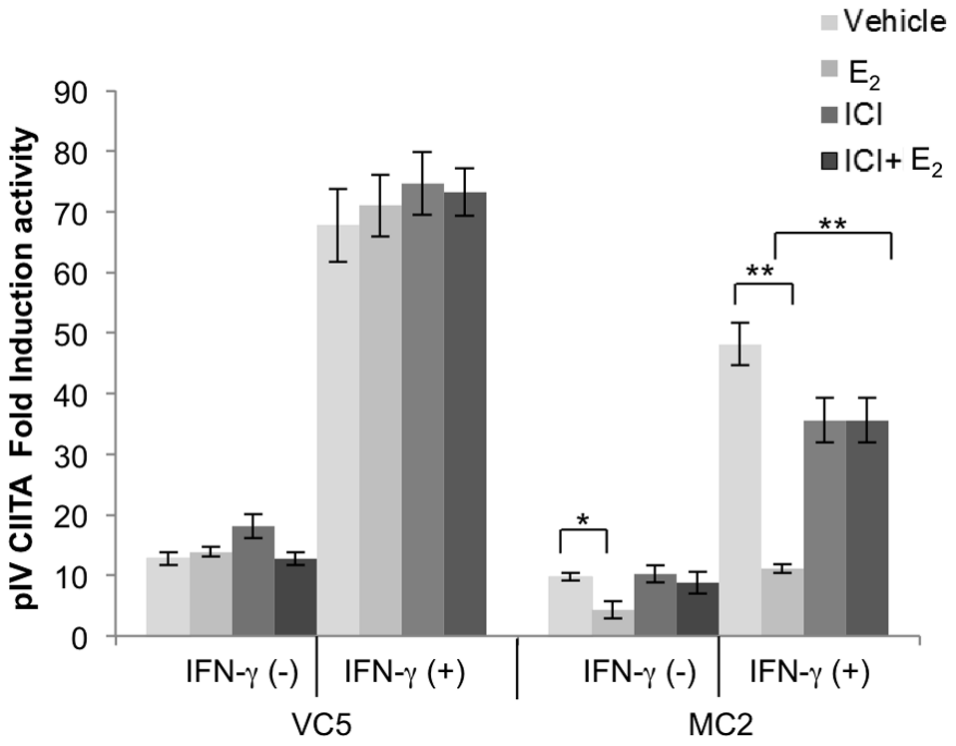
E_2_-ERα signaling pathway interferes with CIITA pIV activity in MC2. VC5 and MC2 cells were cultured in E_2_-depleted media followed by transfection with CIITA pIV luciferase constructs. On the following day, cells were treated with vehicle (ethanol), E_2_ (10^−9^ M) and/or ICI (10^−6^ M), and stimulated or not with IFN-γ (100 U/ml) for 12 hours. Data are expressed as fold induction over the PGL2 Basic empty plasmid after controlling for transfection efficiency using cells dual transfected with GFP (Green Florescent Protein). The effect of ERα on the transcription activation of CIITA PIV was determined from relative luciferase activities in transfected MC2. Error bars represent the mean ± SEM of three independent experiments (**p<0.01).

Since IFN-γ inducible HLA-II expression requires activation of CIITA pIV [33], we hypothesized that E_2_ activation of ERα interferes with CIITA pIV activity. We transfected VC5 and MC2 with a CIITA pIV luciferase construct and treated the cells with E_2_ and/or ICI, followed by stimulation or not with IFN-γ for 12 hours. E_2_-treatment further reduced both basal and IFN-γ induced CIITA pIV activity in MC2, while ICI reversed the inhibitory effect of E_2_ in MC2 cells (Figure 6). Treatment with ICI and/or E_2_ did not significantly affect constitutive or IFN-γ inducible CIITA pIV activity in VC5.

**Figure 7:**
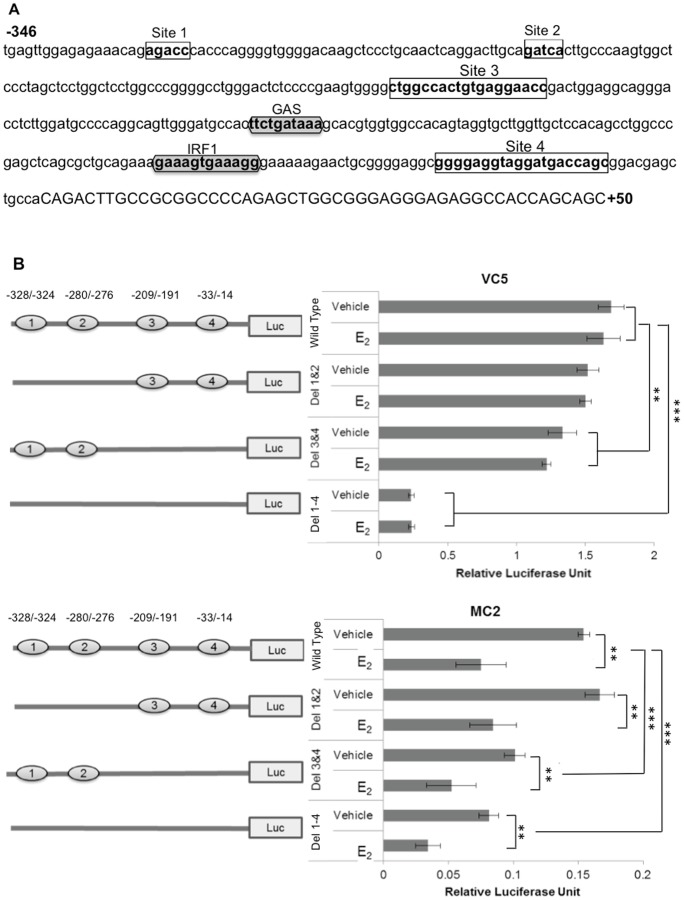
Mutation of putative ERE sites in CIITA pIV does not enhance CIITA pIV activation in MC2. (A) CIITA pIV nucleotide sequence from −346 to +50 with the GAS and IRF1 binding sites (shaded hexagon) and the predicted ERE (clear rectangles) were identified using online transcription factor prediction software, (http://tfbind.hgc.jp/, http://alggen.lsi.upc.es/ and http://www.cbrc.jp/index.eng.html). Site directed mutagenesis was used to perform deletion of the predicted ERE. (B) VC5 and MC2 were transfected with CIITA pIV constructs, then treated with vehicle (ethanol) or E_2_ (10^−9^ M) and stimulated with IFN-γ (100 U/ml) for 12 hours, followed by determination of luciferase activity. Bar graphs represent the mean ± SEM of three independent experiments (**p<0.01, ***p<0.001).

To determine whether E_2_ directly regulates CIITA pIV activity, we searched for presence of ERE sites using three different computer software programs (http://tfbind.hgc.jp/, http://alggen.1si.upc.es/ and http://www.cbrc.jp/index.eng.html) and identified four putative ERE sites in CIITA pIV (Figure 7A, bold letters in boxes). Sites 1 to 3 are upstream of the STAT1 and IRF1 binding sites. Site 4 is downstream of these sites and precedes the start codon. To determine if either of these sites serves as an ERα repressor of CIITA transcription, three deletion mutant constructs (Site 1/2 deletion mutant, Site 3/4 deletion mutant and Site 1–4 deletion mutant) were created (Figure 7A, open boxes). VC5 and MC2, transfected with one of the mutant CIITA pIV constructs, were pretreated with E_2_ or vehicle control and then stimulated with IFN-γ for 12 hours, followed by measurement of luciferase activity (Figure 7B, left panel). All three deletion constructs demonstrated significantly reduced IFN-γ stimulated CIITA pIV activity in E_2_-treated MC2, similar to that observed in MC2 transfected with the wild type CIITA pIV plasmid. By comparison CIITA pIV activity was similar in E_2_ or vehicle treated VC5 cells whether transfected with wild type or deletion constructs. Intriguingly, constructs Del 3 & 4 and Del 1–4 resulted in dramatic and significant loss of CIITA pIV activity in both cell lines, suggesting there may be other or overlapping sites in CIITA pIV that interact with currently unknown transcription factors for a fully active promoter. Alternatively, the deletion of these sites may have led to the creation of a novel site that has an inhibitory effect on CIITA pIV activity. Importantly, these results do not support the hypothesis that diminished CIITA pIV activity in MC2 treated with E_2_ occurs via ERE sites in the proximal region of CIITA pIV.

### E_2_-ERα interferes with STAT1 signaling in ERα transfected MC2 cells

**Figure 8:**
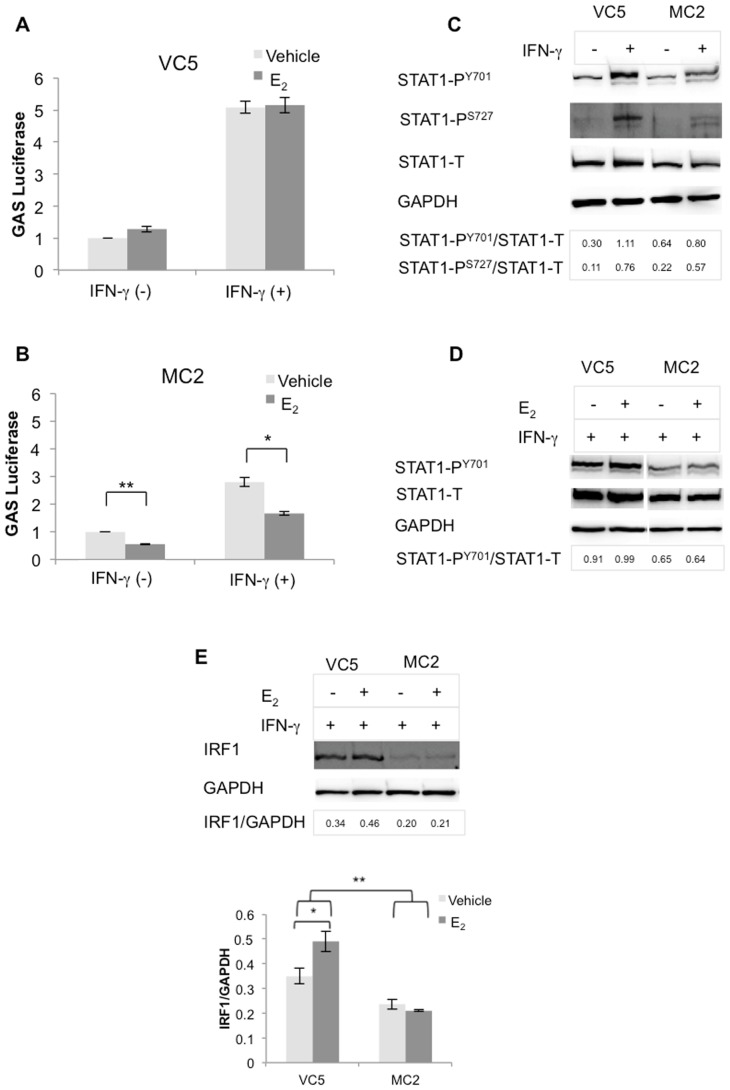
GAS promoter activity, STAT1 activation and IRF1 expression were reduced in MC2 as compared to VC5. (A) VC5 and (B) MC2 were cultured in E_2_-depleted media and transfected with 8 X GAS binding sequence construct, then treated with vehicle (ethanol), E_2_ (10^−9^ M) and stimulated or not with IFN-γ (100 U/ml) for 6 hours. Firefly luciferase activities in samples were normalized to Renilla luciferase activities in the same samples and expressed as fold induction over the un-stimulated mock. Error bars represent the mean ± SEM of three independent experiments (*p<0.05, ** p<0.01). (C) VC5 and MC2 were stimulated with IFN-γ (100 U/ml) for 15 minutes, STAT1 activation was detected using STAT1 Phospho-Tyrosine^701^ and Phospho-Serine ^727^ antibodies. (D) VC5 and MC2 were treated or not with E_2_ (10^−9^ M) for 4 hours, followed by stimulation with IFN-γ (100 U/ml) for 15 minutes, STAT1 activation was detected using STAT1 Phospho-Tyrosine^701^. (E) Western blot analysis of whole cell lysates, prepared from VC5 and MC2 stimulated with IFN-γ (100 U/ml) for 96 hours, for IRF1 (BD-20) expression. Error bars represent the mean ± SEM of three independent experiments (*p<0.05, *** p<0.001).

To explore whether STAT1 signaling, necessary for activation of CIITA pIV, is adversely affected by ERα activation, we transfected the 8 X GAS luciferase plasmid in VC5 and MC2, followed by treatment, or not, with E_2_ and/or IFN-γ for 6 hours. Compared to VC5, STAT1 signally was clearly reduced in MC2 (Figure 8A & 8B); moreover, E_2_ significantly reduced basal and induced GAS promoter activity by about 44% and 40%, respectively, in MC2 (Figure 8B). Although E_2_ increased basal GAS promoter activity by about 28% in VC5, this was not significant; E_2_ had no effect on induced activity (Figure 8A).

To test whether reduced GAS activity in MC2 was the result of reduced pSTAT1, we performed Western blot analysis on lysates from cells treated or not with IFN-γ for 15 minutes. As shown in Figure 8C, total STAT1 and pSTAT1 at tyrosine (Y) 701 and serine (S) 727 were reduced in MC2, compared to VC5. Similar results were observed in an experiment in which cells were also treated with E_2_ for 4 hours, followed by IFN-γ treatment for 15 minutes; moreover, E_2_ did not alter levels of phosphorylated or total STAT1 in MC2 or in VC5 (Figure 8D). We next examined IRF1 expression, also essential for CIITA pIV activation, in MC2 and VC5, treated with E_2_ and stimulated with IFN-γ for 96 hours (Figure 8E). We found IRF1 levels were significantly decreased in MC2, compared to VC5, that E_2_-treatment had only a trivial effect on IRF1 in MC2, whereas it significantly increased the levels in VC5. Collectively, these results show that ectopic expression of ERα and, moreover, its activation by E_2_ attenuates STAT1 signaling, however, E_2_ has only a marginal inhibitory effect on IRF1 levels in MC2. These findings imply that attenuation of CIITA pIV and subsequent reduced HLA-II expression in ERα positive breast cancer may be due to defects in STAT1 regulation.

### E_2_ differentially affects IFN-γ signaling in established ERα^+^ and ERα^−^ breast cancer cells

**Figure 9:**
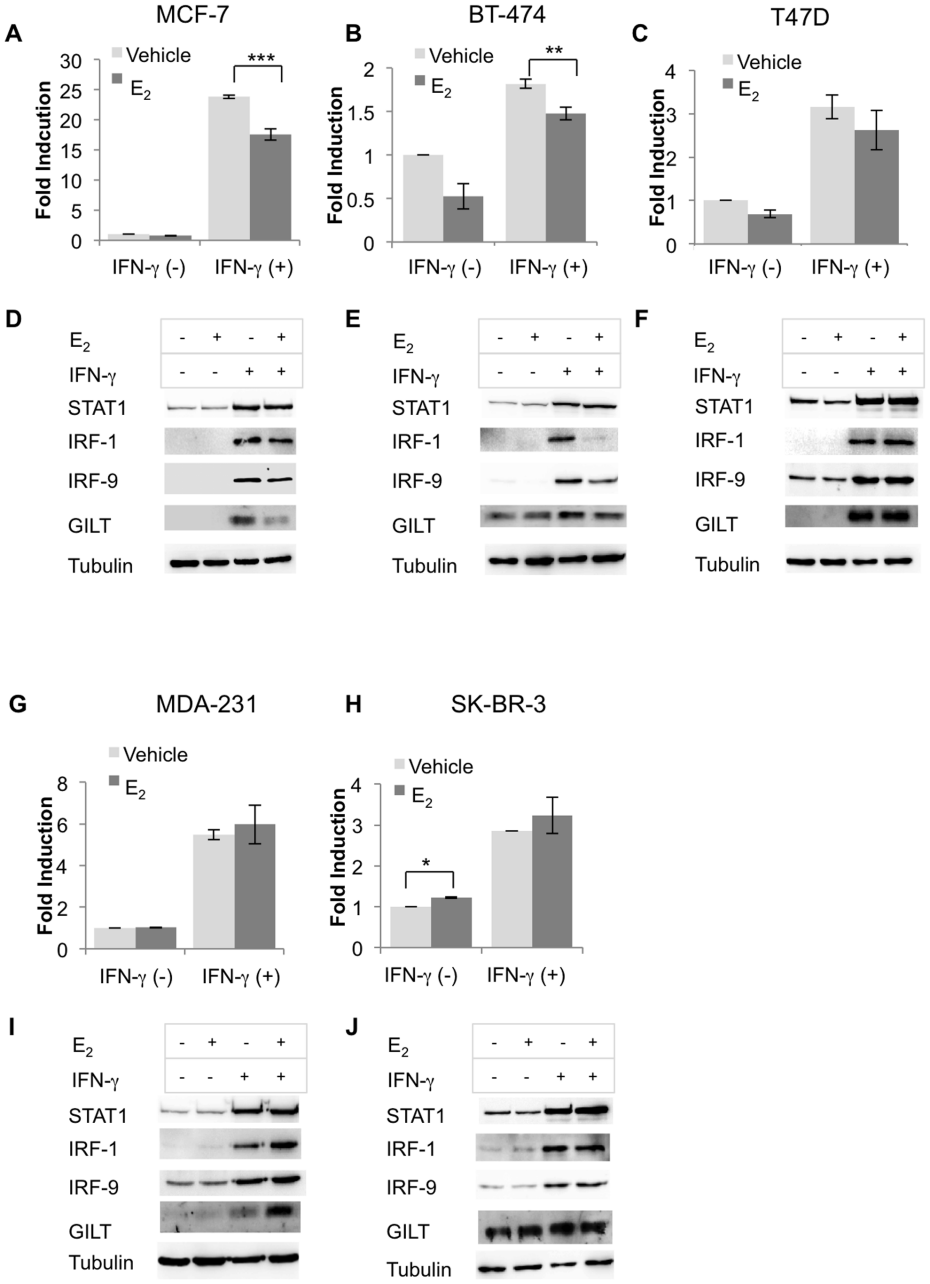
E_2_ differentially down regulates IFN-γ signaling and IFN-γ induced proteins in endogenous ER^+^ breast cancer cell lines. (A) MCF-7, (B) BT-474, (C) T47D, (G) MDA-MD-231, and (H) SK-BR-3 were cultured in E_2_-depleted media, transfected with 8 X GAS binding sequence construct, then treated with vehicle (ethanol), E_2_ (10^−9^ M) and stimulated or not with IFN-γ (100 U/ml) for 6 hours. Firefly luciferase activities in samples were normalized to Renilla luciferase activities in the same samples and expressed as fold induction over the un-stimulated mock. (D) MCF-7, (E) BT-474, (F) T47D, (I) MDA-MB-231 and (J) SK-BR-3 were cultured in E_2_-depleted media, treated with vehicle (ethanol), or E_2_ (10^−9^ M) and stimulated or not with IFN-γ (100 U/ml) for 96 hours. Western blot analysis of cytoplasmic extracts was performed for expression of IFN-γ inducible proteins: STAT1 (06-501), IRF1 (BD-20), IRF9 (C-20), GILT (T-18). Each figure represents one of three independent experiments.

To ensure that attenuated STAT1 signaling in MC2 was not merely a peculiarity of the transfected model, we further analyzed GAS promoter activity in endogenously ERα^+^ BCCL: MCF-7, BT-474 and T47D and ERα^−^ BCCL: MDA-MB-231 and SK-BR-3. E_2_ significantly decreased IFN-γ induced GAS activity in MCF-7 and BT-474, (Figure 9A & 9B) but not in T47D (Figure 9C). To further confirm the inhibitory effect of E_2_ on IFN-γ signaling in BCCLs, other than HLA-DR (Figure 1), we conducted Western blot analysis of IFN-γ inducible proteins. These included STAT1, IRF1, IRF9, a member of the IRF family of transcription factors that is not implicated in CIITA expression [51], and gamma-interferon-inducible lysosomal thiol reductase (GILT), a STAT1 regulated but CIITA-independent protein, that is important for antigen processing [52] Basal and IFN-γ inducible STAT1 levels were not substantially altered by E_2_ in either cell line (Figure 9D–9F); however, STAT1 regulated proteins, IRF1, IRF9 and GILT were differentially modulated in E_2_-treated MCF-7 and BT-474 (Fig 9D & 9E).

In contrast to the E_2_-inhibitory effect on GAS promoter activity in the ERα^+^ lines, E_2_ noticeably enhanced GAS promoter activity in ERα^−^ BCCL, MDA-MB-231 and SK-BR-3 (Figure 9G & 9H). Furthermore, E_2_-treatment augmented expression of IRF1 and GILT in MDA-MB-231 cells, and of STAT1 in SK-BR-3 (Figure 9I & 9J). Taken together, the results suggest that E_2_ differentially modulates the IFN-γ and HLA-II pathways in ERα^+^ and ERα^−^ BCCL.

## Discussion

We previously reported the frequency of HLA-II positive tumor cells in ER^+^ breast carcinomas is decreased, compared to ER^−^ tumors from younger women [12]. As estrogen levels are high in breast carcinoma tissues, irrespective of age and menopausal status[41], we hypothesized a negative role for estrogen-activated ERα in HLA-II regulation in breast cancer cells. Herein, we provided experimental evidence that ERα and E_2_-activated ERα attenuate HLA-II expression in BCCL. Using paired ERα (MC2) and vector (VC5) transfected MDA-MB-231 clone 10A cells we showed: i) E_2_-treatment coordinately decreased IFN-γ inducible HLA-II and CIITA in ERα^+^ MC2 but not in ERα^−^ VC5; ii) reduction of ERα by ICI or siRNA reversed the E_2_-inhibitory effect on HLA-II expression, CIITA pIV activity and transcriptional activation of CIITA in MC2; iii) E_2_-activated ERα adversely affected IFN-γ induced transcription as shown by GAS reporter assay and expression levels of IFN-γ inducible proteins. Importantly, similar results were observed in the ERα^+^ BCCL, MCF-7 and BT-474, in which GAS activity, STAT1 regulated genes and HLA-DR were down regulated by E_2_; by contrast, E_2_ augmented GAS activity and expression of STAT1 regulated genes in the ERα^−^ BCCL, MDA-MB-231 and SK-BR-3.

Overall our data support a negative role for E_2_-ERα signaling in the regulation of HLA-II in breast cancer cells, but cell-specific differences are evident. For example, E_2_ treatment attenuated HLA-DR in MCF-7 and BT-474, but not in T47D. This finding is compatible with an older study in which BCCL, cultured in E_2_-sufficient medium, exhibited a hierarchy of IFN-γ inducible HLA-DR levels with T47D>MCF-7>BT-474 [6]. Differential HLA-II in these cells is not surprising, given that ER^+^ BCCL, although expressing many of the same genes associated with a luminal subtype, will differ in expression of many other genes [53], which may or may not be regulated by E_2_. Multiple factors including the ratio and localization of ERα and ERβ receptors, levels of coactivators and corepressors, cell surface receptors such as GPR30 and EGFR and cross-talk with other signaling pathways determine which genes are up or down regulated [54]. E_2_-activated ERβ inhibits recruitment of ERα to ERE in target genes, thus, suppressing ERα regulated gene expression [55]. Furthermore, activation of the ERβ2 isoform results in ERβ2/ERα heterodimers that are targeted for proteasomal degradation [56]. It is noteworthy, then, that E_2_ increases ERβ in T47D but not in MCF-7 or BT-474 [57] and the ER β:α ratio in T47D is reported to be greater than in MCF-7[53], [58] thus, suggesting that cell-specific differences in ER subtypes and other receptors may underlie differential HLA expression in breast cancer.

The most convincing evidence that activated ERα modulates HLA-II and CIITA expression came from our experiments using the transfected ERα^+^ line, MC2. Since MC2 and its ERα^−^ vector control, VC5, are derived from MDA-MB-231 clone 10A, which is negative for both ERα and ERβ[47], it should be a valid model to directly assess the effect of activated ERα on the HLA-II pathway. Our finding, that E_2_ attenuation of HLA-II and CIITA in MC2 could be reversed by knockdown of ERα in MC2 with ICI (Figures 3D and 4A) or siRNA (Figures 5A), provides compelling evidence that the classical ERα signaling pathway interferes with CIITA regulation. However, we were puzzled that even without adding E_2_, HLA-II and CIITA were reduced in MC2 and that knockdown of ERα by ICI and siRNA did not restore CIITA activity in MC2 to VC5 levels. Although we used phenol red free medium and E_2_-depleted FBS, there might still be a minimum level of E_2_ in the culture medium, which is sufficient to activate ERα and suppress CIITA activity. Furthermore, the incomplete depletion of ERα by ICI or siRNA (Figures 3D, 4A & 5A), may also explain why HLA-II and CIITA expression were not completely restored.

Identification of putative ERE binding sites in the proximal region of CIITA pIV (Figure 7A) led us to explore a direct role for ERα as a suppressor of CIITA pIV activation. Although mutagenesis of these sites did not reverse the inhibitory effect of ERα or E_2_-activated ERα on CIITA pIV activity (Figure 7B), the experiments do not completely exclude direct ERα suppression of CIITA activity as there may be other unidentified ERE sites in either the proximal or distal region of CIITA pIV through which this effect is mediated. Alternatively, ERα may indirectly suppress CIITA pIV activation through interacting with another factor such as AP1 or NFKβ that may bind CIITA pIV [21], or by interacting with factors such as CREB, SRC-1 and CBP/p300 [59] that interact with the regulatory elements of CIITA pIV and HLA-II promoters [23], [60], [61]. This remains to be further studied.

Although others have shown an E_2_ inhibitory effect on MHC class II expression [17], [19], [44], [45], the described mechanisms were not CIITA dependent. Tzortzakaki et al (2003) reported E_2_-inhibition of IFN-γ inducible HLA-DR in both MCF-7 and T47D, whereby the mechanism involved sequestering the steroid receptor co-activator 1 (SRC-1) away from the HLA-DRA promoter by the E_2_-activated ER [17]. Our study did not assess cofactors, but similarly, we found E_2_-inhibition of DR expression and DRA promoter activity with only slightly reduced CIITA in MCF-7 (Figure 1 and data not shown). However, our results for T47D conflict with theirs, as we found no E_2_ inhibition of HLA-DR in this cell line. This could be due to differences in the amounts of E_2_, as their study used 3–4 log fold more than ours. Higher than physiological concentrations of E_2_ were also used to show an E_2_ inhibitory effect on murine MHC-II that did not involve reduced CIITA[45]. Here the E_2_ inhibitory effect was mediated through reduced association of the histone acetylation transferase, CBP, with the MHC-II promoter. Since CBP is required for acetylation of histones 3 and 4 in the MHC-II promoter, this resulted in decreased transcription of MHC-II. Intriguingly, the cell lines in this study expressed both ER subtypes, which bound to the MHC I-Eβ promoter, but as neither ICI nor tamoxifen reversed the E_2_ inhibitory effect on MHC-II promoter, they concluded the mechanism was ER-independent. Subsequently, they showed the E_2_ inhibitory effect on CBP was mediated through E_2_ activation of JNK MAPK pathway [45]. Although these studies are not directly comparable to ours, they do suggest additional factors may have contributed to E_2_-inhibition of HLA-DR. However, the underlying mechanisms for E_2_-ERα inhibition of CIITA transactivation and STAT1 signaling in breast cancer are likely to be more diverse and complex.

Studies investigating deficient CIITA and MHC class II expression in various cancer cell lines have identified epigenetic modifications that result in transcriptional silencing [61], [62]. These include histone deacetylation of the CIITA pIV in squamous cell carcinomas [63] and rhabdomyosarcomas [64], and hypermethylation of the CpG islands in CIITA pIV colon and gastric carcinoma lines. Hypermethylation and recruitment of dysregulated methyltransferases were hypothesized as mechanisms for defective CIITA and HLA-II expression in metastatic breast cancer [65], [66], but these studies were based on a presumed breast cancer cell line MDA-MB-435. This cell line and its metastatic variants have a controversial history [67], as there is strong evidence that they originated from a melanoma cell line [68]. However, it is conceivable that epigenetic modifications are implicated in the E_2_-liganded ERα deleterious effect on CIITA pIV, as numerous epigenetic modifications have been described in breast cancer that include silencing of ERα in the MDA-MB-231 cell line and downregulation of tumor suppressor genes [69]–[73].

In our study the E_2_ mediated downregulation of CIITA pIV and HLA-II expression in the ERα^+^ BCCL appears likely due to aberrant STAT1 signaling with reduced expression of IRF1 or reduced ability to bind the CIITA promoter. Others have shown that STAT1 and IRF1 are aberrantly expressed in some ER^+^ breast cancer tissues and cell lines [74]–[77] and both have tumor suppressor properties. Chan et al (2012) reported significantly decreased STAT1 in human neoplastic tissue of ER^+^ breast tumors and showed that knocking out STAT1 in a mouse model correlated with the development of ER^+^PR^+^ luminal A adenocarcinoma [77]. Intriguingly, the reduced phosphorylation of STAT1 and reduced levels of total STAT1 in MC2, compared to VC5 (Figure 8C), whether treated or not with E2 (Figure 8D) implies that ERα somehow negatively regulates STAT1 activation and signaling. We speculate this could occur via direct interaction of ERα with STAT1, possibly interfering with dimerization and nuclear translocation or indirectly by interfering with STAT1 promoter activation. Whatever the mechanism, aberrant STAT1 signaling is likely to result in reduced IRF1 levels and subsequently reduced CIITA activation. However, as ICI treatment of MC2 did not substantially increase STAT1 levels (data not shown), nor completely degrade ERα, more studies are required to test this concept.

A potential explanation for the dramatic reduction of CIITA pIV activity in MC2 is decreased IRF1 (Figure 8D), which is essential for IFN-γ inducible CIITA transcriptional activation and HLA-II expression [50], [78], [79]. Furthermore, E_2_ diminished IRF1 in MCF-7 and dramatically reduced its expression in BT-474, a cell line that expresses insignificant amounts of HLA-DR in the presence and absence of E_2_ (Figures 1 & 9). In contrast, ERα^−^ lines appear to have an intact IFN-γ signaling pathway that is not inhibited by E_2_. We did not investigate mechanisms underlying E_2_-mediated increase in GAS and STAT1 activity, but others have shown a dependency on SRC kinase activity [80]. Furthermore, E_2_ also activates other pathways such as MAPK and PI3K pathways that interact with the JAK-STAT1 pathway [40], [81], [82].

In conclusion, our results show that HLA-II expression is regulated differently by estrogen in ER^−^ and ER^+^ breast cancer cells. To our knowledge this report is the first to show that activation of ERα by its ligand E_2_, results in downregulation of CIITA pIV activity. Although the mechanism is not fully elucidated, the data suggest that the dysregulation occurs at the level of STAT1 activation. Such a mechanism would explain the HLA-DR negative tumor cells in breast carcinomas despite infiltrating T-cells and high levels of IFN-γ and has further implications for tumor immune escape.

## Materials and Methods

### Cells

Breast cancer cell lines, obtained from ATCC, included: ERα^+^ (MCF-7, T47D, and BT-474) and ERα^−^ (SK-BR-3, MDA-MB-231 (MDA-231). Cells were grown in Iscove's Modified Dulbecco's Medium (IMDM) (Gibco) supplemented with 10% heat inactivated fetal bovine serum (FBS) (Gibco), 2 mM L-glutamine, antibiotic-antimycotic mixture (100 units/ml penicillin G sodium, 100 µg/ml streptomycin sulfate, and 0.25 µg/ml amphotericin B as Fungizone®), all from Invitrogen. MDA-MB-231 clone 10A and two stably-transfected lines, MC2 (MDA-MB-231 clone 10A transfected with *ESR1* (*NM_000125*) and VC5 (MDA-MB-231 clone 10A transfected with an empty vector) were generous gifts from Dr. Craig Jordan. Cells were grown in phenol red free minimum essential medium (MEM) (Invitrogen) supplemented with 5% charcoal/dextran heat inactivated FBS (CD FBS) (Hyclone), MEM non-essential amino acids, 6 ng/ml recombinant human insulin, 2 mM L-glutamine and antibiotic-antimycotic mixture (all from Invitrogen). MC2 and VC5 were maintained under selective conditions with G418, 5 µg/ml (Sigma). For experiments cell lines were detached with 0.25% trypsin (Invitrogen) and plated at 3×10^5^ cells/well in 6-well plates or 2×10^4^ cells/well in 96-well plates. After 24 hours medium was replaced with fresh medium containing 10^−9^ M E_2_ and/or 10^−6^ M ICI (Sigma) or vehicle control (ethanol) and left un-stimulated or stimulated with IFN-γ, 100 units/ml (BD Biosciences) for the indicated times depending on the experiment.

### Antibodies

Expression of HLA-II and CIITA was determined as follows: HLA-DR conformers, clone L243 [83] ATCC, purified IgG2a from supernatant diluted to 2.4 µg/ml for flow cytometry (FC) or 10 ng/ml for Western blot analysis; HLA-DRα, mouse IgG1 (clone Tal 1B5, Abcam, 40 ng/ml, IB); Ii, mouse IgG1 (clone LN2, BD Biosciences, 5 µg/ml, FC or 200 ng/ml, IB); HLA-DM, mouse IgG1 (clone MaP.DM1, BD Biosciences, 10 µg/ml, FC and clone TAL18.1, Abcam, 40 ng/ml, IB); CIITA (rabbit antiserum # 21, diluted 1/4000), prepared in Dr. Viktor Steimle's laboratory [84]. Other antibodies used for Western blotting included anti-ERα, rabbit IgG (HC-20, Santa Cruz Biotechnology, 500 ng/ml); STAT1, rabbit IgG (06-501, Upstate Biotechnology, 200 ng/ml); STAT1 Phospho-Tyrosine^701^ and Phospho-Serine ^727^, both rabbit IgG (GenScript, 500 ng/ml); ISGF-3γ p48 (IRF9), rabbit IgG (C-20, Santa Cruz Biotechnology, 400 ng/ml); IRF1, mouse IgG1 (clone BD-20, BD Biosciences, 125 ng/ml); GILT, goat polyclonal IgG (T-18, Santa Cruz Biotechnology, 250 ng/ml). Isotype-matched nonspecific monoclonal antibodies (mAbs) included: IgG2a (clone NSG2a) from a local source and IgG1 (clone MOPC-21, BD Biosciences). Housekeeping proteins were detected with anti-GAPDH, mouse IgG1 (clone 6C5, Abcam, 1 ng/ml); α-tubulin, mouse IgG1 (clone B-7, Santa Cruz Biotechnology, 250 ng/ml) and anti-nuclear matrix protein p84, mouse IgG2b (clone 5E10, Abcam, 1 µg/ml). Horse Radish Peroxidase (HRP)-conjugated affiniPure F(ab)_2_ fragment goat anti-mouse (GAM) IgG, Fc specific and HRP-conjugated affiniPure F(ab)_2_ fragment goat anti-rabbit (GAR) IgG, Fc specific antibodies, were purchased from Jackson Immunoresearch and HRP conjugated donkey anti-goat (DAG) antibody IgG, was purchased from Santa Cruz Biotechnology.

### Flow cytometry

Flow cytometry was performed as previously described [85]. Briefly, trypsin-harvested cells, 2×10^5^ cells/tube, were incubated with 25 µl of appropriate mAbs in wash buffer (0.2% CDFCS, 0.02% NaN3 in PBS) for 30 minutes at 4°C. Antibody binding was detected with phycoerythrin (PE) labeled goat anti-mouse (GAM) conjugate (Jackson Immunoresearch), followed by fixation in 1.0% paraformaldehyde (PFA) and analyzed using a FACS Calibur flow cytometer (Becton-Dickinson). For intracellular staining, the cells were fixed in 2% PFA and permeabilized with 0.2% Tween 20 in PBS (Sigma) prior to adding primary antibodies, diluted in wash buffer containing 0.2% Tween 20 and 0.5% BSA.

### Western Blotting

Nuclear and cytoplasmic extracts were prepared using Nuclear Extract Kit (ActiveMotif) according to the manufacture's protocol. Whole cell lysates (WCL) were prepared in either Triton X-100 buffer (PBS pH 7.4,Triton X-100 1%, 0.5 M ethylene-diaminetetraaccetic acid) or RIPA buffer (PBS, pH7.4, 1% NP40, 0.5% sodium deoxycholate, 0.1% sodium dodecyl sulfate) containing protease inhibitors aprotinin (1 µg/ml), leupeptin (1 µg/ml), pepstatin A (1 µg/ml) and phenylmethylsulfonyl fluoride (10 µg/ml). Proteins, quantified using a BCA protein assay kit (Thermo-Fisher Scientific), were reduced with 2-mercaptoethanol and electrophoresed (10 µg/lane) using 8–10% SDS PAGE, followed by western blotting. Membranes, treated with blocking buffer (5% milk powder in TBS-Tween (0.15 M NaCl, 0.05 M Tris pH 7.4, 0.05% Tween 20) for 1 hour, were incubated overnight with primary antibodies at 4°C. Antibody binding was detected with appropriate HRP-conjugated secondary antibodies and Immobilon Western Chemiluminescent HRP substrate (Millipore). Immunoreactivity was visualized and quantified by scanning densitometry using ImageQuant LAS 4000 and ImageQuant TL8.1 software, respectively (GE Healthcare).

### Real-time RT-PCR

Total RNA, extracted using TRIzol Reagent (Invitrogen) and treated with Ambion® TURBO™ DNase to remove contaminating DNA, was quantified using NanoDrop (Thermo Scientific). The High Capacity cDNA Reverse Transcription kit (Applied Biosystems) was used for cDNA synthesis according to the manufacturer's protocol. Real time PCR was performed using TaqMan® Probe-Based Gene Expression Analysis kit for CIITA (Hs00172106_m1) and GAPDH (Hs99999905_m1) following the manufacturer's recommendations. Quantification was performed by the comparative threshold cycle (ΔΔ^CT^) method and normalized to GAPDH using StepOnePlus™ (Applied Biosystems). A control sample without RNA and a reference sample (RAJI, B cell line) were included in each experiment.

### siRNA Transfection

Cells, plated in a 6-well plate at 3×10^5^ cells/well for 24 hours, were transfected with either 25 nM ON-TARGET plus SMART pool siRNA for *ESR1* or non-targeting siRNA (Dharmacon, USA) using 4 µl DharmaFECT4 transfection reagent (Dharmacon, USA) per well according to the manufacturer's protocol. Forty-eight hours later, the cells were treated with E_2_ 10^−9^ M or vehicle control (ethanol) and stimulated with IFN-γ, 100 units/ml, for 4 or 24 hours for mRNA and protein expression, respectively.

### Reporter gene assays

The CIITA promoter IV firefly luciferase construct [79] and the 8 X GAS firefly luciferase construct [86] were kind gifts from Dr. Jenny Ting and Dr. Eleanor N. Fish, respectively. Transfection conditions were optimized using Fugene HD (Roche) transfection reagent according to the manufacturer's protocol: briefly a master mix was prepared by diluting the appropriate plasmid with Opti-MEM (Gibco) to a concentration of 0.02 µg/µl; Fugene HD was added to the same mixture in the ratio of 7∶2 (Fugene HD in µl:Plasmid DNA in µg) and left for 20 minutes at ambient temperature. Cells, plated in a 96-well plate at 2×10^4^ cells/well for 24 hours, at 37°C were transfected with 5 µl of this mixture and incubated for an additional 24 hours. The medium was then replaced with medium containing the appropriate treatments and incubated for 12 hours for CIITA pIV or 6 hours for 8 X GAS constructs. Transfection efficiency was estimated by co-transfecting the cells with SV-40 Renilla luciferase or green fluorescent protein (GFP). Luciferase activity was measured using the dual luciferase assay system (Promega) and a 96-well luminometer (Fluoroskan Ascent Fl, Labsystems).

### Generation of CIITA pIV deletion constructs

Different sets of deletion mutants of P-346/+50 CIITA pIV were generated by site-directed mutagenesis using QuikChange Lightning Site-Directed Mutagenesis Kit (Stratagene) according to the manufacturer's instructions. Mutagen primers (Table 1) were designed with Agilent's web-based QuikChange Primer Design Program Sequences. Sequences, deleted from the original template, are bolded and underlined. All deletions were confirmed by sequencing.

**Table 1:** Mutagen primers used to generate the CIITA PIV deletion constructs

**Site 1**	Original template	ctcaacctctctttgtc**tctgg**gtgggtccccacccctg
Primers	Del −328/−324 Fw	5′-ttggagagaaacagcacccaggggtggg-3′
	Del −328/−324 Rv	5′-cccacccctgggtgctgtttctctccaa-3′
**Site 2**	Original template	gacgttgagtcctgaacgt**ctagt**gaacgggttcaccgaggga
Primers	Del −280/−276 Fw	5′-caactcaggacttgcacttgcccaagtggctc-3′
	Del −280/−276 Rv	5′-gagccacttgggcaagtgcaagtcctgagttg-3′
**Site 3**	Original template	agaggggcttcacccc**gaccggtgacactccttgg**ctgacctccgtccctg
Primers	Del −209/−191 Fw	5′-ccccgaagtgggggactggaggcagg-3′
	Del −209/−191 Rv	5′-cctgcctccagtcccccacttcgggg-3′
**Site 4**	Original template	cttgacgcccctccg**cccctccatcctactggtcg** cctgctcgacggtgt
Primers	Del −33/−14 Fw	5′-ctgcggggaggcggacgagctgcc-3′
	Del −33/−14 Rv	5′-ggcagctcgtccgcctccccgcag-3′

### Statistics

Statistical analysis was performed using Microsoft excel 2010 software. One-way analysis of variance (ANOVA) and Tukey post hoc tests were used for comparisons within a group. The student t-test was used for comparing two different treatments for one cell. All tests were two-sided and p<0.05 was considered significant.
